# Avatar therapy for bothersome tinnitus using an augmented reality mobile application: a pilot feasibility study

**DOI:** 10.3389/fdgth.2026.1787660

**Published:** 2026-08-03

**Authors:** Kaho Magami, Hiroaki Yata, Yasuo Kabe, Sho Kanzaki, Ken Hayashi, Hirokazu Takahashi

**Affiliations:** 1 Graduate School of Information Science and Technology, The University of Tokyo, Tokyo, Japan; 2 Department of Otolaryngology, School of Medicine, Keio University, Tokyo, Japan; 3 Department of Otorhinolaryngology, Kamio Memorial Hospital, Tokyo, Japan; 4 International Research Center for Neurointelligence, The University of Tokyo, Tokyo, Japan

**Keywords:** subjective tinnitus, phantom sensation, augmented reality, digital health intervention, avatar-based therapy

## Abstract

Digital avatar-based interventions have been proposed as a promising approach for managing phantom sensations such as tinnitus by externalizing the perceived source of sensation and enabling users to interact with it. However, the emotional impact of symptom-representing avatars on users has received limited attention; it remains unclear whether emotional closeness with avatars influences impressions of phantom sensations and subjective symptom experiences. This pilot study aimed to explore the feasibility and emotional impact of an augmented reality (AR)-based avatar therapy for subjective bothersome tinnitus. We developed a mobile AR application that represents tinnitus as interactive avatars, allowing users to engage with them in everyday environments. Participants reported transient alleviation of symptoms immediately following application use, as well as a nominal improvement in tinnitus-related impression after the 1-week intervention period. Exploratory analyses suggested a possible association between depressive/anxiety-related stress responses and short-term changes in tinnitus botheration. This pilot evaluation provides preliminary insights into the feasibility and emotional aspects of AR-based avatar therapy for tinnitus. The findings inform the design of future controlled studies and suggest that emotionally oriented interactions with symptom-representing avatars may represent a promising direction for digital health interventions targeting tinnitus and other phantom sensations.

## 1 Introduction

Phantom sensations, such as tinnitus and phantom limb pain, emerge without any external physical stimuli. A putative common mechanism underlying these sensations is predictive coding (1–5), a framework in which the brain continuously generates top-down predictions about incoming sensory input and updates them based on bottom-up prediction errors, or discrepancies between expected and actual input (6). Under normal conditions, spontaneous neural activity is suppressed because the brain's dominant prediction favors silence; prediction errors arising from such activity are deemed uninformative and discarded. However, when this prediction is shifted, or when the precision weighting assigned to prediction errors is increased by factors such as deafferentation, stress, or excessive attention, residual spontaneous activity can no longer be effectively suppressed. This results in persistent, unresolved prediction errors that the brain interprets as meaningful sensory input, giving rise to phantom sensations (2, 5). Moreover, the unexpected and uncontrollable nature of these sensations frequently elicits negative emotional responses and heightened attention to such sensations, which may contribute to symptom exacerbation through a maladaptive feedback loop between phantom sensation and negative emotion (7–12).

Virtual reality (VR)-based interventions have been proposed as a promising approach for managing phantom sensations. Such interventions aim to externalize the source of physically non-existing sensations by projecting them onto avatars in a virtual environment and allowing users to interact with or manipulate them (13–20). For example, in VR therapy for phantom limb pain, attempts to move the phantom limb are coupled with movements of a virtual limb, which has been associated with reduction in pain following repeated training (14, 18). In the context of tinnitus, VR-based approaches have projected tinnitus sounds onto avatars that participants could spatially manipulate, with reported changes in tinnitus perception (17, 19, 21). These approaches primarily focus on enhancing a sense of controllability over phantom sensations.

However, in these conventional VR-based interventions, the potential emotional impact of interacting with symptom-representing avatars, such as the development of attachment or emotional closeness, has received relatively limited attention. This is despite evidence that interactions with VR avatars can foster emotional engagement and closeness (22, 23) and may exert beneficial effects in mental health contexts, including depression and borderline personality disorder (24–26). From a digital health perspective, it remains critical to evaluate not only symptom outcomes but also human factors such as emotional engagement, acceptability, and individual psychological characteristics that may moderate intervention efficacy in real-world settings.

Given that distorted impressions of phantom sensations, shaped by a built-up feeling of uncontrollability and negative counseling provided by specialists (e.g., “nothing can be done to tinnitus”), are considered to contribute to symptom deterioration (8–10, 12), we hypothesize that the emotionally positive interaction with symptom-representing avatars may help shape better impression of phantom sensations and potentially influence on symptom alleviation. The present pilot study therefore aimed to explore the feasibility and emotional impact of an avatar-based intervention for subjective bothersome tinnitus, a condition in which negative emotional responses to phantom sounds are thought to contribute to symptom exacerbation and chronicity (8–10). We developed a mobile augmented reality (AR) application that represents tinnitus as an interactive avatar, enabling users to engage with their perceived sound in everyday environments. The application attempts to improve the impression of tinnitus through positive interactions with the tinnitus avatar and to alleviate the symptom by weakening the negative loop between the impression and the phantom sensation. In addition, given the frequent association between tinnitus and depressive symptoms (27–30), we exploratorily examined whether individual differences in depressive and anxiety-related responses to stress were associated with observed changes in symptom severity following the intervention.

## 2 Materials and methods

The primary outcomes of this pilot study were feasibility and changes in tinnitus-related impressions, reflecting a human-factor–oriented evaluation of the digital health intervention in real-world use.

### 2.1 Compliance with ethical standards

All procedures were approved by the Ethics Committee of Kamio Memorial Hospital, and written informed consent was obtained from all participants. The authors declare no conflicts of interest.

### 2.2 Participants

Participants were patients with subjective bilateral tinnitus who were undergoing outpatient treatment at a tinnitus clinic. Individuals with severe hearing loss or unstable psychological conditions were excluded. Of the 15 patients recruited, 12 participated in the study (8 females; mean age = 47.6 years, SD = 9.4). Three participants withdrew prior to study completion, reporting that they found the application uninteresting or unconvincing.

### 2.3 Overview of the mobile application

A mobile AR application was developed using the Unity 3D game development platform (Unity Technologies) to provide a positive interactive experience with a virtual representation of tinnitus. Participants completed questionnaires before and after gameplay (see Section 2.4). The entire session lasted approximately 5 min.

#### 2.3.1 Interaction game

**Figure 1:**
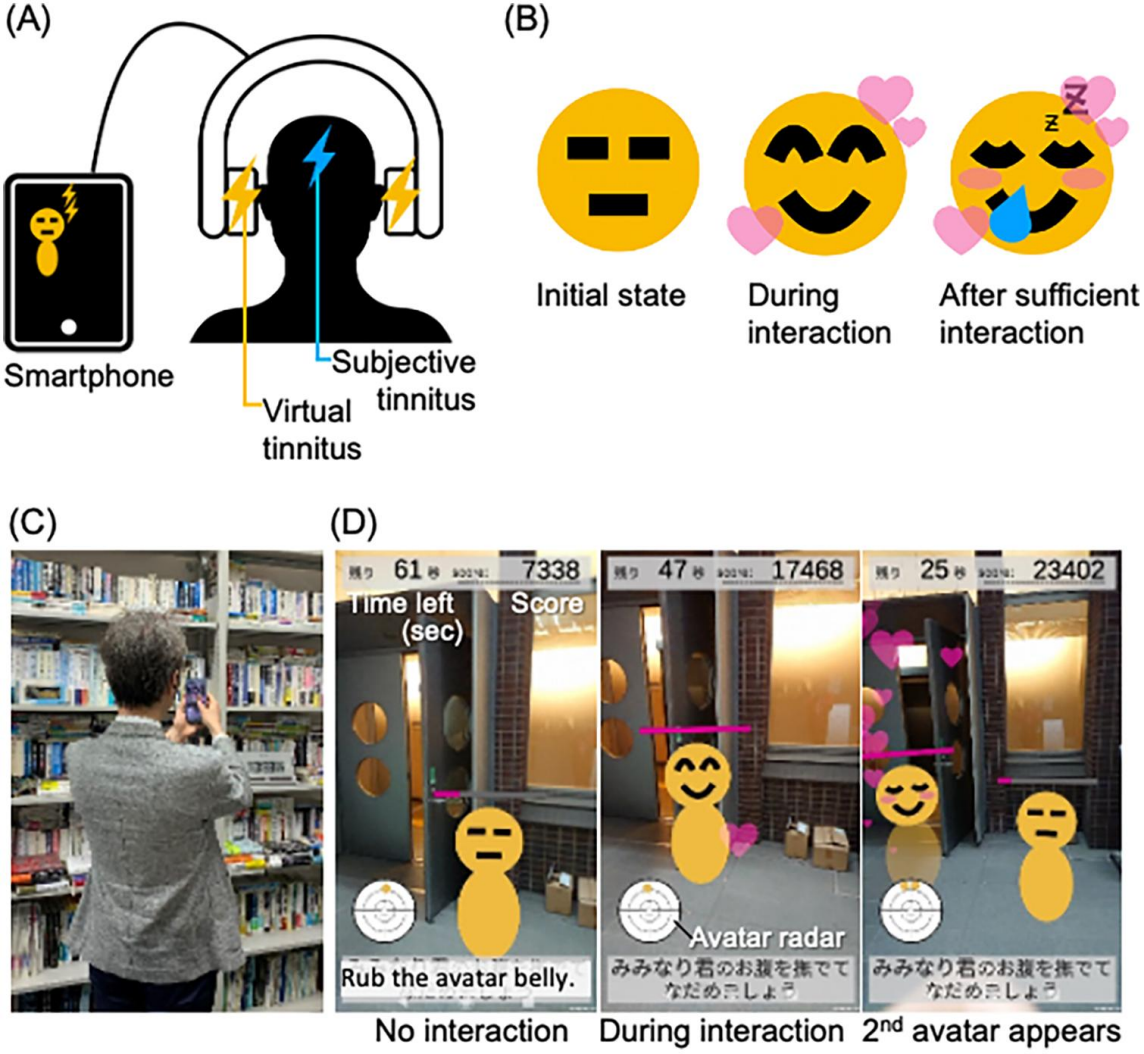
Overview of the mobile application. **(A)** System overview. **(B)** Facial expressions of the tinnitus avatar. **(C)** Gameplay scenery. **(D)** Gameplay screen.

The application featured a tinnitus avatar designed to facilitate emotionally positive interactions with virtual tinnitus. The avatar, which emitted a pseudo-tinnitus sound (see Section 2.3.2) via earbuds, moved within the AR space and displayed a facial expression (Figure 1A). Participants were instructed to rub the avatar's belly for as long as possible. Positive feedback was provided through changes in the avatar's facial expression (from neutral to smiling), the appearance of heart symbols, and a gradual reduction in the volume of the virtual tinnitus sound (Figure 1B). After a randomly determined duration of continuous interaction (uniformly distributed between 6 and 9 s), the avatar fell asleep, accompanied by a visual sleep cue (Figure 1B), and the sound ceased. This interaction was intended to reduce negative impressions associated with tinnitus. To maintain engagement, a new avatar emitting the same virtual tinnitus sound could appear after the previous avatar fell asleep. If a sleeping avatar was left unattended for more than 30 s, it reactivated and resumed sound emission. The design was intended to allow multiple cycles of emotionally positive experiences within a single session. During gameplay, avatar locations were displayed on a radar interface beneath the AR screen (Figure 1D). After the session, participants were presented with the following message to reinforce the intended framing of the interaction: “Tinnitus has no intention to bother you. Please try to maintain a positive relationship with tinnitus, as you did in the game.”

#### 2.3.2 Virtual tinnitus sound

The effectiveness of the avatar-based intervention depends on the extent to which the virtual avatar can be assimilated to each participant's subjective tinnitus. Prior to gameplay, participants customized the virtual tinnitus sound to approximate their own tinnitus by adjusting sound type, frequency, and volume. Sound type was selected as either a pure tone or 1/3-octave band noise. Frequency was adjustable between 125 and 12,000 Hz, and sound level was set to subjectively match the participant's tinnitus loudness. For safety, the playback volume during gameplay was automatically reduced by 30% relative to the selected level. The customized virtual tinnitus was spatialized by manipulating interaural level differences via stereo earbuds, enabling the sound to appear spatially linked to the visual avatar as it moved within the AR environment.

### 2.4 Procedure

Pure-tone audiometry (500, 1,000, 2,000, and 4,000 Hz) was conducted using a clinical audiometer to measure the pure-tone average. Participants then completed questionnaires at the hospital, including the Stress Response Scale (SRS-18), which assesses stress responses (31), the Tinnitus Handicap Inventory (THI), which measures severity of tinnitus distress (32), and a custom impression questionnaire assessing subjective impressions of tinnitus using a 7-point Likert scale (Table 1A). SRS-18 and THI were administered once at baseline and were not re-administered following the intervention. The impression questionnaire was designed based on typical counseling advice used in educational therapy for bothersome tinnitus in our clinics, such as framing tinnitus as non-harmful and encouraging a non-confrontational attitude toward the symptom. Participants were also given an opportunity to practice using the application and to ask questions at the hospital.

**Table 1:** Questionnaires for the system evaluation.

(A) Impression questionnaire	
Items	7-Point Likert scale
Q1 What is your impression of your tinnitus?	Enemy—friend
Q2 How do you feel when you perceive your tinnitus?	I want to eliminate it—I do not mind it
Q3 How do you respond to your tinnitus?	I want to punish it—I leave it alone
Q4 How would you like to deal with your tinnitus?	I want to eliminate it—I want to let it go

(B) Perception questionnaire	
Items	Visual analog scale (0–10)
Q5 Loudness of perceived tinnitus	Low–high
Q6 Botheration level of perceived tinnitus	None–extreme
Q7 Severity of perceived tinnitus	None–severe

Participants were subsequently loaned a smartphone (Xperia XZ1, Sony) and stereo earbuds (HSE-A1000, ALPEX) to use the application at home. They were encouraged to use the application once a day for one week. No reminders were provided during this period. Gameplay logs (e.g., played dates, total play time, interaction duration, and customized sound parameters) were automatically recorded. Before and after each gameplay session, participants completed an in-app perception questionnaire. Based on the tinnitus rating and severity scales, this questionnaire assessed (1) tinnitus strength/loudness, (2) level of botheration, and (3) perceived severity (Table 1B). Each item was rated on a visual analog scale ranging from 0 to 10. At the end of the 1-week intervention, participants again completed the custom impression questionnaire (Table 1A) to assess changes in subjective impressions of tinnitus.

### 2.5 Statistical analysis

Statistical analyses were conducted to evaluate changes in tinnitus-related impressions and perceptions before and after the intervention. Normality of questionnaire scores was assessed using the Shapiro–Wilk test. For comparisons where normality was not violated, paired *t*-tests were conducted. For measures violating normality assumptions, Wilcoxon signed-rank tests were applied. In addition, linear mixed-effects models were fitted using the *lme4* package in R (33), with participant as a random effect and sex, age, THI score, and number of gameplay sessions, and duration of outpatient treatment as fixed effects, to account for individual and demographic variability. Given the small sample size, mixed-effects models were conducted as exploratory sensitivity analyses and were considered supplementary to the primary paired *t*-test/Wilcoxon signed-rank test results. Kendall's rank correlation was used to explore associations between pre-post differences in perceptual measures and (1) the pre-post difference in tinnitus impression and (2) participants' depression/anxiety level. *P*-values were corrected for multiple comparisons using the Holm method.

## 3 Results

### 3.1 Participant characteristics and application usage

For the 12 participants included in the analyses, the threshold of the pure-tone average across 0.5–4 kHz were 15.1 ± 10 dB HL (mean ± SD) in the right ear and 13.4 ± 10.6 dB HL in the left ear, corresponding to normal hearing levels or, in some cases, mild hearing loss. During the smartphone loan period, participants used the AR application an average of 8.1 ± 5.5 times, with a mean play duration of 110 ± 39 s per session.

### 3.2 Impression of tinnitus before and after the intervention

**Figure 2:**
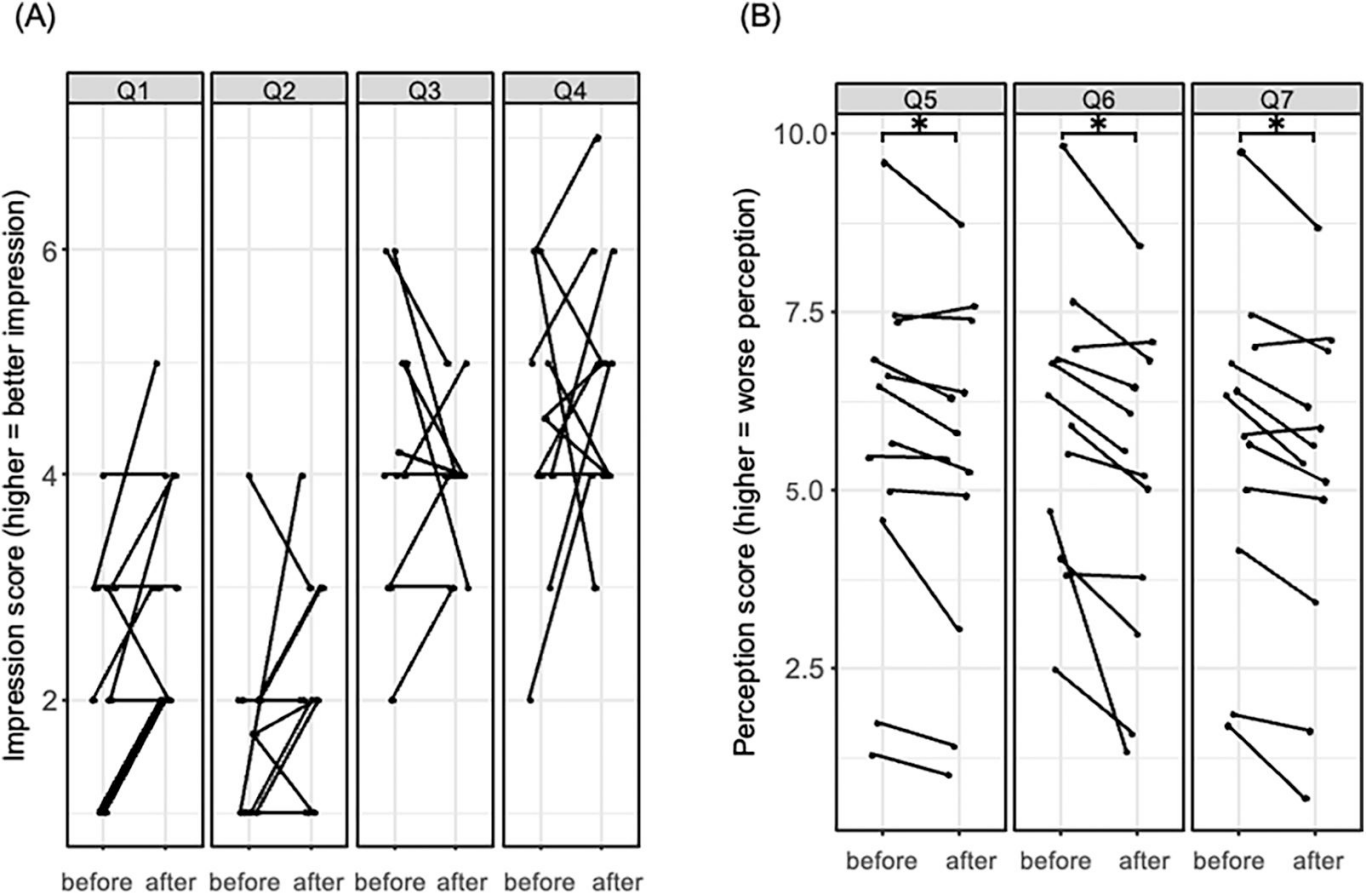
Effects of the application on the tinnitus-related impression and perception scores. **(A)** Changes in tinnitus-related impression scores after the 1-week intervention. Higher values indicate more positive impressions. Each line represents the score change for an individual participant. Question numbers correspond to the items listed in Table 1A. **(B)** Perception scores before and after gameplay. Higher values indicate worse tinnitus perception. Each dot represents the mean score across gameplays for each participant, and each line indicates the score change for an individual participant. Question numbers correspond to the items listed in Table 1B. *p < 0.05 after correction for multiple comparisons.

The effect of the game on tinnitus-related impressions was assessed by comparing scores on the impression questionnaire (Table 1A) obtained before and after the 1-week intervention period. For Q2–Q4, two participants (subjects 8 and 9) did not provide responses before the intervention; these missing values were replaced with the mean score of the remaining participants. Figure 2A shows individual pre- and post-intervention scores for Q1–Q4. The paired *t*-test revealed a nominal improvement in Q1 (“Enemy–Friend”) following the intervention [*t*_(11)_ = −2.60, *p* = 0.02, Cohen's *d* = −0.75], suggesting a possible shift in tinnitus-related impression following the intervention; however, this did not survive multiple comparison correction across Q1–Q4 (corrected *p* = 0.10) and should be interpreted as exploratory. No significant changes were observed for the remaining items [Q2: *t*_(11)_ = −1.55, *p* = 0.15, *d* = −0.45; Q3: *t*_(11)_ = 0.94, *p* = 0.37, *d* = 0.27; Q4: *t*_(11)_ = −0.76, *p* = 0.46, *d* = −0.22; all corrected *p* > 0.45]. A supplementary mixed-effects analysis accounting for demographic variability (see Methods) yielded a consistent pattern.

### 3.3 Immediate change in tinnitus symptoms

We reasoned that improvements in tinnitus-related impressions following the intervention might be accompanied by short-term perceptual changes through a weakening of the negative loop between impression and tinnitus perception. Table 2 summarizes the tinnitus perception before and after gameplay for each participant. Because responses to some items violated normality assumptions, Wilcoxon signed-rank tests were used. Significant reductions were observed in tinnitus loudness (Q5: *V* = 74, *p* = 0.003, *r* = 0.60), botheration level (Q6: *V* = 76, *p* = 0.001, *r* = 0.65), and perceived severity (Q7: *V* = 75, *p* = 0.002, *r* = 0.62), all of which remained significant after multiple comparison correction across Q5–Q7 (corrected *p* = 0.005, 0.004, and 0.005, respectively) (Figure 2B). A supplementary mixed-effects analysis accounting for demographic variability yielded consistent results across all three measures (Q5: *b* = 0.43, SE = 0.13, *p* = 0.001; Q6: *b* = 1.01, SE = 0.17, *p* < 0.001; Q7: *b* = 0.60, SE = 0.13, *p* < 0.001; all corrected *p* < 0.01). These findings are consistent with the hypothesis that short-term improvements in tinnitus perception may co-occur with changes in tinnitus-related impressions.

**Table 2:** Perception questionnaire scores and duration of outpatient treatment for each participant.

Subject	Q5 Loudness		Q6 Botheration		Q7 Severity		Duration of outpatient treatment (weeks)
	Before	After	Before	After	Before	After	
1	5.26 (1.28)	5.70 (1.13)	6.57 (0.91)	6.06 (2.20)	6.81 (1.89)	5.75 (0.94)	170
2	0.86 (1.11)	0.84 (0.50)	3.87 (4.07)	1.53 (1.03)	1.40 (0.96)	0.75 (0.51)	6
3	6.91 (0.34)	6.26 (0.61)	6.74 (0.24)	5.94 (0.41)	6.82 (0.26)	6.04 (0.66)	31
4	1.85 (1.29)	1.46 (1.10)	2.63 (1.42)	1.58 (0.56)	1.95 (0.92)	1.62 (0.71)	283
5	7.71 (3.63)	6.79 (3.65)	8.43 (3.88)	7.66 (4.31)	7.82 (3.80)	6.67 (3.46)	219
6	4.66 (2.44)	3.18 (1.28)	3.75 (1.91)	2.93 (1.81)	4.61 (2.70)	3.83 (1.06)	5
7	9.86 (0.72)	8.83 (1.00)	10.00 (0.14)	8.54 (2.53)	10.00 (0.14)	8.81 (1.75)	34
8	6.52 (0.32)	6.30 (0.90)	3.40 (0.90)	3.56 (0.83)	5.71 (0.28)	5.75 (0.69)	32
9	5.00 (1.22)	4.96 (0.60)	5.66 (0.99)	5.14 (0.86)	5.05 (0.59)	5.00 (1.29)	42
10	5.67 (0.00)	5.28 (0.00)	5.91 (0.00)	5.03 (0.00)	5.66 (0.00)	5.13 (0.00)	42
11	7.53 (1.33)	7.46 (0.95)	7.56 (1.23)	6.44 (1.81)	7.46 (0.78)	6.81 (2.32)	218
12	7.69 (0.76)	7.39 (0.53)	6.86 (0.44)	7.08 (1.22)	7.61 (1.34)	7.21 (0.71)	138

Responses were collected before and after each gameplay session. Median values and interquartile ranges are shown.

### 3.4 Relationship between perceptual and impression changes

**Figure 3:**
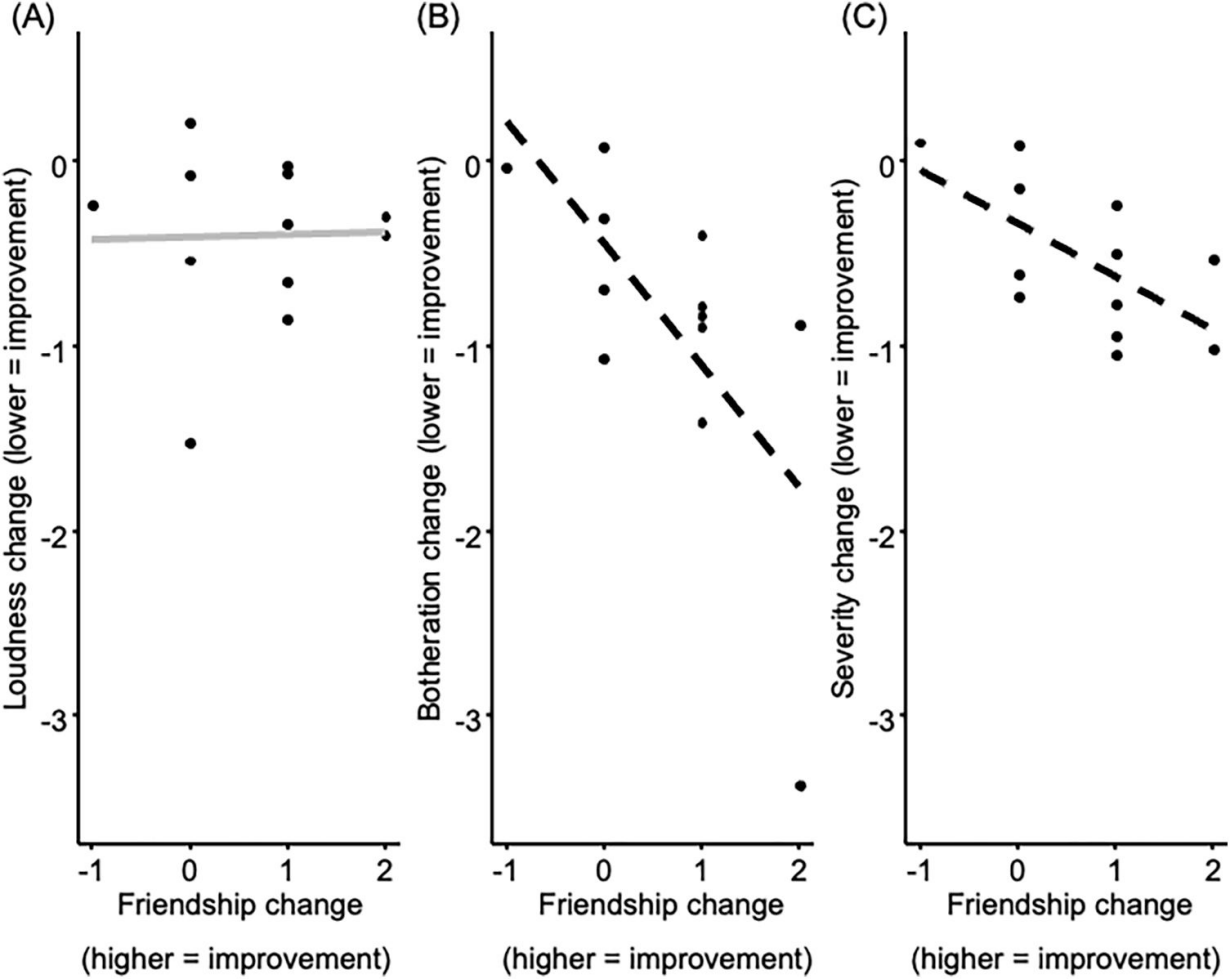
Relationships between changes in the friendship score and immediate perceptual changes (**A**: loudness change, **B**: botheration change, **C**: severity change). Higher values on the *x*-axis indicate improvement in friendship scores, whereas lower values on the *y*-axis indicate improvement in perceptual measures. Each dot represents an individual participant. Dashed regression lines indicate *p* < 0.1, and the grey regression line indicates *p* > 0.1.

To examine potential relationships between changes in tinnitus perception and tinnitus-related impressions, we calculated the pre-post differences for each measure and correlated these values. Three perceptual measures were compared with the impression measure that showed nominal improvement (Figure 3). For the three perceptual measures, a more negative difference indicates a reduction in symptom severity, whereas for the impression measure, a more positive difference indicates an improved impression. As some variables violated normality assumptions and included ties, Kendall's rank correlation was applied. No significant correlation was observed between tinnitus loudness (immediate effect on Q5) and friendship (1-week effect on Q1) (*τ* = −0.05, *p* = 0.83, corrected by the Holm method for multiple comparisons). Moderate correlations were found between botheration level (Q6) and friendship (Q1) (*τ* = −0.55, *p* = 0.02), and between severity (Q7) and friendship (Q1) (*τ* = −0.51, *p* = 0.03), though the significance disappeared after multiple comparison correction (*p* = 0.07). Previous tinnitus studies also failed to improve the loudness component of the symptoms based on the alteration of the impression of tinnitus (21, 34), suggesting a weak relationship between the loudness suppression and the impression change.

### 3.5 Relationship between depression level and the changes in symptom severity

**Figure 4:**
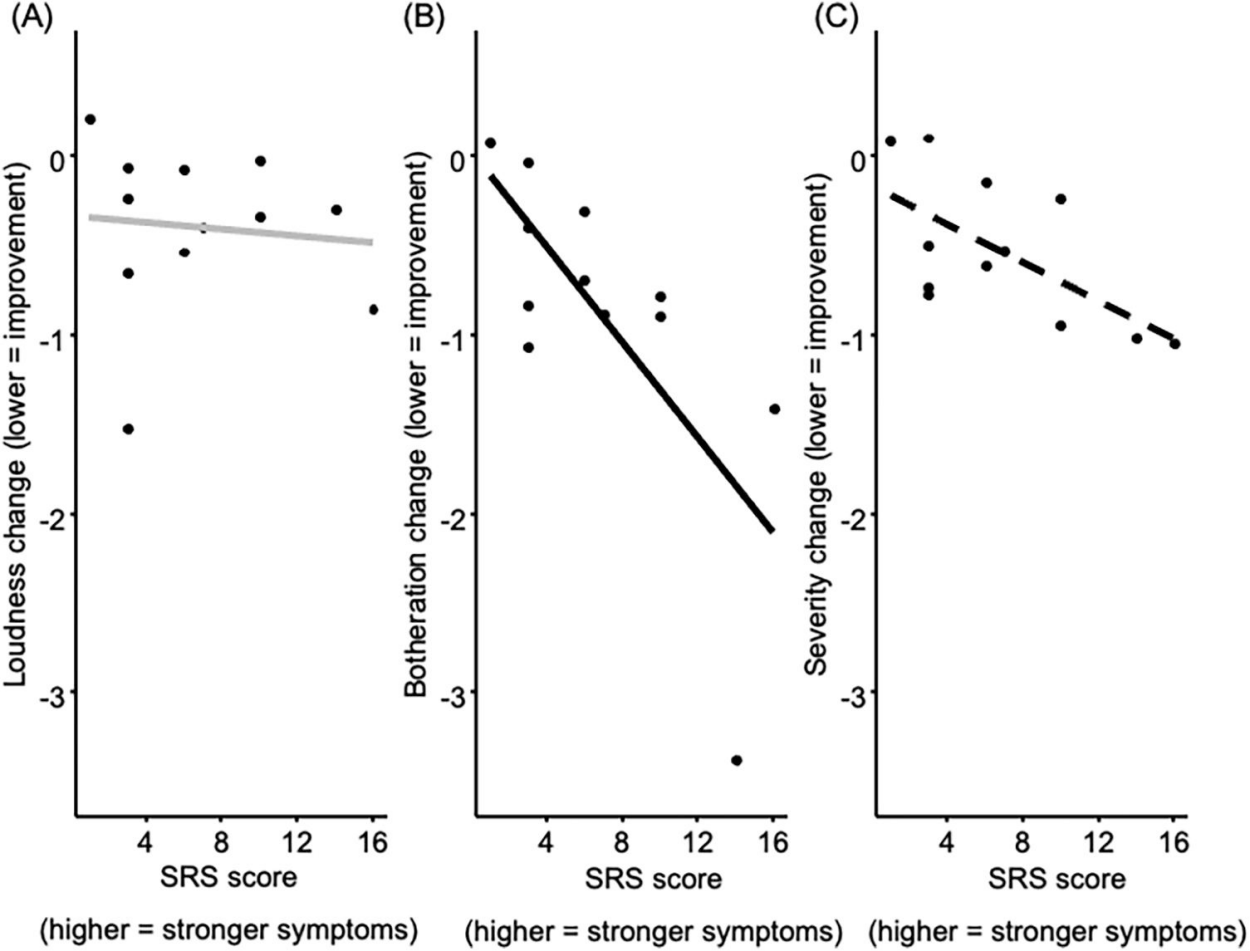
Relationships between the SRS-18 score and the efficacy of the application (**A**: loudness change, **B**: botheration change, **C**: severity change). Higher values on the *x*-axis indicate stronger symptoms, whereas lower values on the *y*-axis indicate improvement in perceptual measures. Each dot represents an individual participant. The black regression line indicates *p* < 0.05, the dashed regression line indicates *p* < 0.1, and the grey regression line indicates *p* > 0.1.

Lastly, we examined whether participants' level of depression/anxiety reaction to stressful events was associated with the changes in symptom severity, defined as changes in perceptual measures. Kendall's rank correlation was used to relate the pre-post differences in the three perceptual measures to the anxiety/depression score of SRS-18. For the perceptual measures, a more negative difference indicates a reduction in symptom severity, whereas for the SRS-18, a higher value indicates a greater tendency toward depression/anxiety. A significant correlation was observed between the SRS-18 score and changes in botheration level (Q6: *τ* = −0.58, *p* = 0.01; after correction: *p* = 0.03), indicating that participants with higher depression/anxiety tended to experience greater reductions in tinnitus botheration. Similarly, a moderate association was found for severity (Q7: *τ* = −0.49, *p* = 0.03; after correction: *p* = 0.07). However, no correlation was observed for loudness (Q5: *τ* = −0.13, *p* = 0.57; Figure 4). Notably, the loudness change rate exhibited a different trend again, suggesting the presence of a different underlying mechanism associated with loudness perception compared to other tinnitus components.

## 4 Discussion

In this study, we developed a mobile application designed to provide tinnitus patients with a friendly interactive experience with tinnitus avatars, aiming to modify tinnitus-related impressions and alleviate tinnitus symptoms. Questionnaire responses showed a nominal shift in tinnitus-related impressions, with participants tending to perceive their tinnitus more as a “friend” than an “enemy” after the intervention; however, this change did not survive correction for multiple comparisons and should be interpreted as preliminary. These exploratory findings raise the possibility that avatar-based interaction may have the potential to modulate the emotional aspect of the phantom auditory sensation, though this requires confirmation in larger studies. Improving the impression of phantom sensations is considered crucial for symptom mitigation, as distorted beliefs about harmfulness and uncontrollability often make the symptom management challenging (8–10, 12). Accordingly, various therapeutic approaches have attempted to modify tinnitus-related impressions, particularly in patients with subjective tinnitus. Cognitive-behavioral therapy (CBT), for example, seeks to associate tinnitus with positive or neutral experiences and to restructure maladaptive beliefs (12, 34). Educational therapies similarly aim to improve patients' understanding of tinnitus mechanisms and to reinforce the notion that tinnitus itself is not inherently harmful (35, 36). Although these approaches have demonstrated beneficial effects (34, 37–39), their implementation typically requires sustained patient engagement and access to trained specialists, which may limit scalability and availability (20, 36, 40, 41). The mobile application developed in the present study addresses some of these limitations by offering an emotionally oriented intervention that does not rely on direct clinician involvement.

Our results also indicated that tinnitus loudness, botheration level, and severity were significantly reduced shortly after the gameplay, with two perceptual components showing mild associations with changes in tinnitus-related impressions. These findings are broadly consistent with our hypothesis that positive interaction with a tinnitus avatar may attenuate negative impressions of tinnitus and, in turn, alleviate tinnitus-related symptoms. Previous studies suggest that when tinnitus perception is accompanied by adverse emotional reactions, limbic and autonomic nervous systems become engaged, leading to heightened distress such as anxiety or panic (9, 29, 35, 42, 43). Activation of these systems is thought to increase activity in auditory processing pathways, thereby suppression of this negative loop plays a crucial role in mitigating the tinnitus symptoms (9, 44). From this perspective, the nominal shift in tinnitus-related impressions observed in this study may reflect a weakening of this maladaptive feedback loop during the gameplay, possibly contributing to short-term perceptual relief. It should be noted, however, that while these reductions were statistically significant, their mean magnitude was modest (approximately 0.4–0.8 points on a 0–10 VAS; Table 2), and a small number of participants showed slight deterioration rather than improvement. We therefore interpret these findings as supporting the feasibility of detecting short-term perceptual shifts with this application, rather than as evidence of clinically meaningful symptom relief; whether such changes accumulate into clinically meaningful improvements remains to be tested in future studies with larger samples and longer follow-up.

In addition to emotional mechanisms, several procedural and perceptual factors may also have contributed to the immediate effects observed. First, the gradual fade-out of the virtual tinnitus sound may itself have produced a transient perceptual effect resembling attenuation of the target symptom. Second, exposure to a tinnitus-like sound may have induced residual inhibition—a phenomenon in which tinnitus is temporarily suppressed following exposure to an acoustically similar stimulus (45). Third, the closing message presented at the end of each session may have influenced participants' subsequent self-reports through response expectancy effects. Finally, demand characteristics—wherein participants may have inferred the application's intended purpose and responded accordingly—cannot be ruled out as a contributor to the observed changes. Separately, focused engagement in the task may also have altered tinnitus perception, as neural processing of task-irrelevant sounds is suppressed under conditions of high perceptual load (46); because the game required the integration of visual, auditory, and motion-related information, such perceptual load may have further attenuated tinnitus perception during gameplay. At present, it is difficult to determine the relative contributions of these factors to the immediate effects of the application. Future studies incorporating a control condition, such as a game lacking positive interaction, would be necessary to clarify the respective contributions of these factors.

The present experiment also revealed moderate associations between depression/anxiety level and short-term changes in some perceptual measures. Specifically, participants who exhibited stronger depressive or anxiety-related responses to stressors tended to show greater short-term reductions in tinnitus botheration, with a similar trend observed for severity. The application developed in this study attached a friendly signal to the tinnitus experience, whereas depressive patients expect fearful emotions following the stressful tinnitus. From the perspective of exposure therapy, a form of CBT, repeated exposure to a stressor followed by an unexpected positive outcome can gradually modify negative emotional associations with that stressor (47, 48). Importantly, the efficacy of exposure-based interventions is known to be enhanced when prediction error is larger, that is, when outcomes deviate more strongly from prior negative expectations (49, 50). Within this framework, participants with higher SRS-18 scores may have experienced larger prediction errors during interaction with the tinnitus avatar, which could be associated with greater short-term perceptual changes, although this interpretation remains speculative given the correlational nature of the data and the limited sample size. Given that depressive reactions are not unique to tinnitus but are also observed in other forms of phantom sensations (43, 51, 52), AR-based avatar interventions may be potentially effective for a broader population experiencing various types of phantom sensations.

Our experiment provided preliminary evidence supporting the feasibility of AR-based avatar therapy targeting subjective tinnitus; however, the present study used a single-arm design because it was intended as an early-stage pilot feasibility study. This design allowed us to evaluate usability, acceptability, and short-term subjective responses before planning a more rigorous controlled trial. However, the absence of a sham or active control condition is a major limitation and prevents us from distinguishing intervention-specific effects from placebo effects, residual inhibition, attentional diversion, sound exposure, and demand characteristics. A controlled design will be essential in future, larger-scale studies to rule out placebo and other non-specific effects. Nevertheless, the observed correlation between changes in symptom severity and participants' depression/anxiety level argues against a purely placebo-based explanation, as there is no clear evidence to suggest that individuals with higher depressive tendencies are more susceptible to placebo effects. Another limitation revealed by this study is that AR avatar therapy may not be acceptable to all patients. Three out of fifteen participants withdrew from the experiment, reporting that they found the game unconvincing and uninteresting compared to mainstream treatment approaches. For example, one participant questioned the meaning of the avatar's smiling expression and heart symbols, and consequently experienced no positive emotional engagement with the game. We believe that the drop-out rate will decrease once the efficacy of this intervention is more clearly demonstrated. A further limitation concerns the outcome measures. As no standardized questionnaire exists for assessing changes in tinnitus perception and emotional impression in this context, we developed custom instruments informed by established tinnitus scales and educational therapy principles; formal validation of these measures remains a task for future work. Additionally, THI was not re-assessed after the intervention, and future studies—particularly those involving longer intervention periods—should incorporate validated outcome measures at both baseline and follow-up. Other limitations include the use of mean imputation for missing values, which, while pragmatic given the small sample size, may not represent the optimal strategy, and the lack of systematic characterization of hearing thresholds at the individually matched tinnitus frequency, which should be assessed in future studies alongside tinnitus pitch and loudness.

Considering these limitations, further studies are required to establish the clinical efficacy of AR-based avatar therapy for phantom sensations and to clarify the underlying neural mechanisms. In particular, beyond the immediate effects observed here, it will be important to examine whether extended use of the application fosters a sense of control over tinnitus, as suggested by previous VR avatar therapies for phantom limb pain (14, 18). Achieving this goal will require the development of a more engaging and sustainable game design that supports repeated use and richer interaction with the tinnitus avatar, which may in turn enhance therapeutic efficacy.

Despite the preliminary nature of this pilot study, the present work highlights the potential of AR-based avatar interventions for phantom sensations. Compared with conventional VR-based approaches requiring head-mounted displays, smartphone-based AR interventions are more accessible and can be embedded in everyday environments. This ecological context may facilitate the transfer of therapeutic practices into daily life. Combined with emerging digital health technologies, such as app-based CBT platforms (53), AR-based avatar therapy represents a promising direction for future intervention development.

In conclusion, the present work has provided preliminary insights into the feasibility of a mobile application for AR avatar therapy targeting tinnitus. Our preliminary findings suggest that emotionally oriented, avatar-based AR interventions may work as a feasible digital health approach by reshaping patients' relationships with persistent symptoms in real-world contexts. The present study was designed as a pilot feasibility study and was not powered to evaluate clinical efficacy, identify responder subgroups, or assess long-term outcomes. Larger controlled studies using validated tinnitus outcome measures and longer follow-up periods will be required to determine the clinical utility of this approach.

## Data Availability

The raw data supporting the conclusions of this article will be made available by the authors, without undue reservation.
